# Calcaneotalotibial arthrodesis by retrograde intramedullary nailing using expert tibia nail for charcot osteoneuropathy of the foot: A case series

**DOI:** 10.1016/j.ijscr.2019.02.035

**Published:** 2019-03-06

**Authors:** I. Oesman, A.R.B. Asdi

**Affiliations:** a Consultant of Foot and Ankle Division, Staff of Department of Orthopaedic and Traumatology, Faculty of Medicine, Universitas Indonesia, Dr Cipto Mangunkusumo Hospital, Jl. Diponegoro No. 71, Jakarta, 10430, Indonesia; b Department of Orthopaedic and Traumatology, Faculty of Medicine, Universitas Indonesia, Dr Cipto Mangunkusumo Hospital, Jl. Diponegoro No. 71, Jakarta, 10430, Indonesia

**Keywords:** Charcot neuroarthropathy, Limb salvage, Calcaneotibiotalar arthrodesis, Retrograde intramedullary nail, ETN, Case report

## Abstract

**Introduction:**

Charcot osteoneuroarthopathy (CN) is a progressive degenerative arthropathy determined by the interaction of neuropathy, osteopaenia and proinflammatory cytokines. The aim of treatment is to maintain the foot on plantigrade position, recover foot deformity, osseous stability, and prevent ulceration. Intramedullary fixation in calcaneotalotibial arthrodesis has been described in promotion of rigid internal fixation with minimal soft tissue violation producing deformity correction, minimal periosteal aggression, vascular damaged and good functional outcome, with less postoperative fusion time and able to achieve fusion of the ankle and the subtalar joint after failed fusion.

**Presentation of case:**

Two patients with charcot foot underwent tibiocalcaneal arthrodesis with retrograde intramedullary technique by Expert Tibial Nail (ETN). The patients are both male 59 years with right ankle deformity 9 months prior to admission and history of trauma and 40 years old with history of Type 2 Diabetes Mellitus. Visual analog scale (VAS), AOFAS and SF score were assessed pre and post operative.

**Discussion:**

A good stability, plantigrade ankle and painless foot on both patients were achieved with a mean score 9 for satisfaction. Pre and 3 months post operative VAS were 4 and 1, AOFAS Scale were 58 and 83, mean of SF-36 were 28.4 and 48.6 for physical condition, 37.3 and 67.2. for mental condition consecutively. No severe postoperative complication were recorded

**Conclusion:**

Tibiotalocalcaneal arthrodesis with retrograde intramedullary technique using ETN system was proven to have a good option for ankle joint salvage with improvement of clinical and functional score. Hence, its application in the charcot joint is promising.

## Highlights

• Retrograde IM Nailing is a promising therapy for Charcot osteoneuroarthopathy.
• Assessment using VAS, AOFAS Scale, and SF-36 showed good result.
• No complication were recorded with this procedure using Expert Tibia Nail.
• Overall patients satisfaction for this procedure was 9/10.

## 1 Introduction

Charcot osteo-neuroarthropathy (CN) of the foot might induce severe instability and deformity to the ankle joint leading to a substantial disability or even amputation [1,2], The prevalence of Charcot arthropathy ranges from 0 0.1% to as high as 13%. Charcot fractures that are not identified and treated properly may progress to marked joint deformity and to skin ulceration over a bony prominence [3].

Treatment of CN is aimed to maintain or recover foot deformity on plantigrade position, achieve osseous stability, and prevent ulceration in which non-operative measures are often disappointing [2,4]. In a severe deformity or when the subtalar joint is affected, a talectomy and calcaneotibial arthrodesis are to be considered [5].

We reported two cases of male with charcot osteoneuropathy performed retrograde intramedullary nail using Expert Tibia Nail (ETN^®^) by Synthes. After surgery we found a painless, stable ankle with good fixation and corrected deformity.

## 2 Methods

This paper has been reported in line with the PROCESS criteria [6]. We reported a case of fifty-six years old man with right ankle deformity since 9 months prior admission. Five years ago he had a motorcycle accident but didn’t go to doctor and decided to medicate himself by drinking OTC (over-the-counter) medicines, several massages therapy and alternative medicine. Over the period of 4 years, his right ankle was deforming, appearing reddish, swollen, and less sensation gradually before one year ago went to an orthopaedic surgeon and was diagnosed with charcot foot and was performed triple arthrodesis of the ankle. However, since 9 months ago patient started to feel his ankle was deforming again and folded inward but he did not stop his activity. The deformity was getting more severe until he decided to consult again then referred to our institution. Patient has the history of hyperuricaemia, hypercholesterolaemia, hypertension but no diabetic mellitus.

**Fig. 1:**
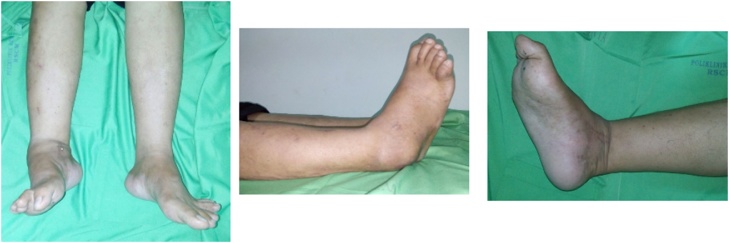
Physical examination of the first case. On the physical examination, there were supinatus and adductus deformity of right foot, minimal swelling, scar and no redness nor sinus.

From physical examination, we found supinatus and adductus deformity of right foot, minimal swelling, scar and no redness nor sinus. (Fig. 1). In palpation, the patient felt tenderness with VAS 2–3, distal sensory is decreased with score 1 on L4-L5-S1, capillary refilling time less than 2 s and limited range of motion of ankle.

**Fig. 2:**
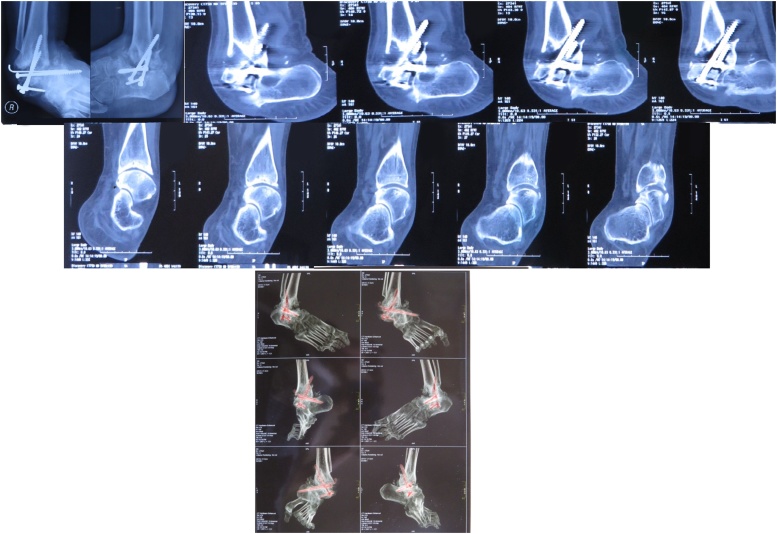
Computed Tomography (CT) Scan examination of the right foot of the first case. Destruction of tibia-fibula distal bone, talus, calcaneus and tarsal bone was observed. Hyperthrophy of metatarsal 1–5, pin on tibia with penetration soft tissue, narrowing of ankle joint, soft tissue oedema which consistent with a charcot arthropathy of right ankle-foot with pin penetration soft tissue.

In laboratory investigations we obtained hyperuricaemia, hypercholesterolaemia and normal blood glucose. From the X-ray anteroposterior and lateral of the ankle we obtained a progessive resorption of talar bone on posterior and subtalar joint, implant loosening and soft tissue oedema. From the Computed Tomography (CT) Scan examination of the right foot, destruction of tibia-fibula distal bone, talus, calcaneus and tarsal bone was observed. Hyperthrophy of metatarsal 1–5, pin on tibia with penetration soft tissue, narrowing of ankle joint, soft tissue oedema which consistent with a charcot arthropathy of right ankle-foot with pin penetration soft tissue (Fig. 2).

**Fig. 3:**
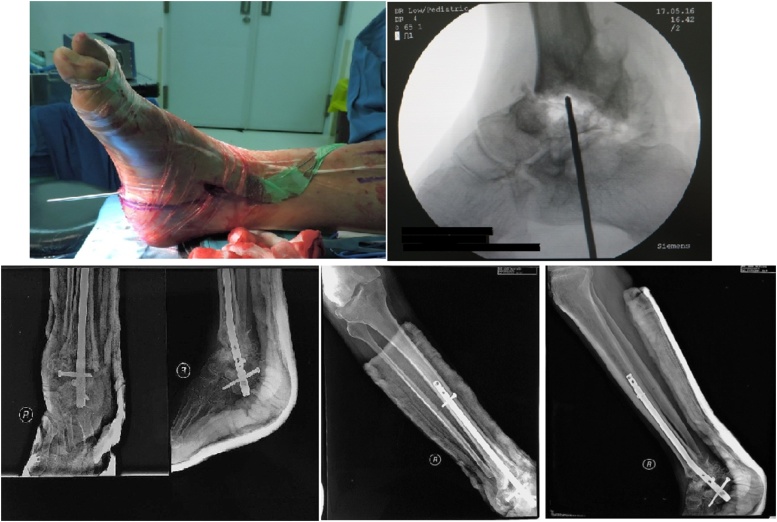
Arthrodesis of CN of the foot by IM nailing with ETN in the first case. After arthrodesis, we corrected the deformity with osteotome os talus on medial and lateral side, and also Achilles tendon lengthening. The joint surfaces of the talus and distal tibia were decorticated. The ankle was brought on fusion position, then we made incision 2 cm on heel to guide wire insertion from distal side of calcaneus through talus, and its position confirmed radiologically. We performed the stability test to prove the arthrodesis, and we got the arthrodesis of the ankle achieved.

From clinical and radiological examination, we confirmed the diagnosis implant failure post arthrodesis of right ankle due to charcot foot neuropathy type 2. This failure was caused by patient non-compliance. We performed implant removal, arthrodesis with Expert Tibial Nail (ETN), bone graft. After that we corrected the deformity with osteotome os talus on medial and lateral side, and also Achilles tendon lengthening to release the stiff joint. The joint surfaces of the talus and distal tibia were decorticated. The ankle was brought on fusion position, then we made incision 2 cm on heel to guide wire insertion from distal side of calcaneus through talus, and its position confirmed radiologically. We performed the stability test to prove the arthrodesis, and we got the arthrodesis of the ankle achieved (Fig. 3).

**Fig. 4:**
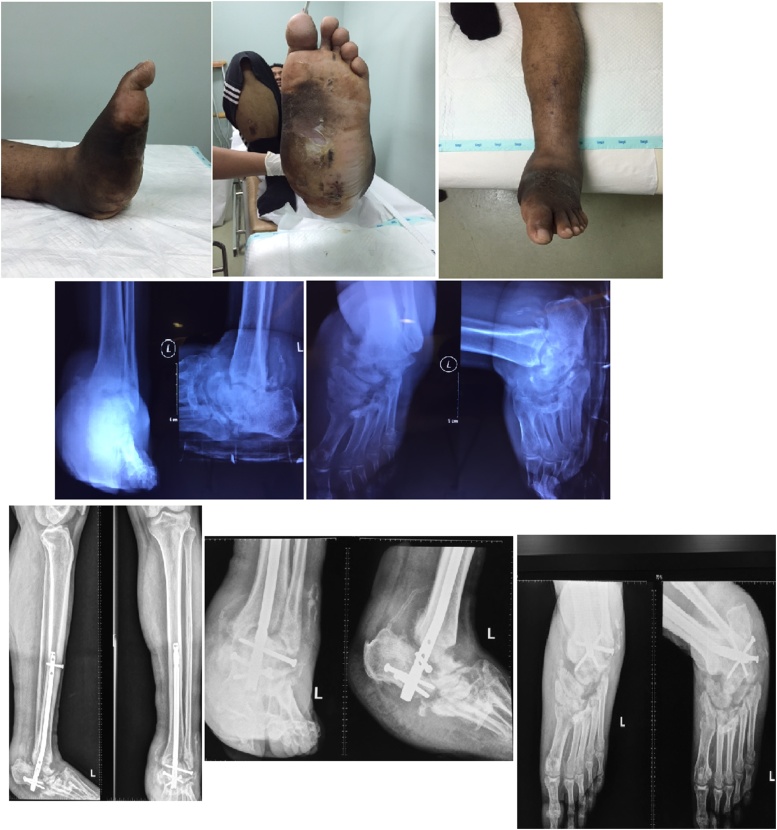
Physical examination and radiography of second case. (A) Physical examintions and (B) radiography showed rocker bottom deformity of the left foot. (C) Radiograpy of the left foot after CCT arthrodesis using ETN.

Second patient was a male 40 years old with uncontrolled and long standing Type 2 Diabetes Mellitus presenting with a rocker bottom deformity of the left foot performed the CCT arthrodesis using ETN (Fig. 4). This patient was prescribed glimepiride and metformine but did not consume routinely.

**Fig. 5:**
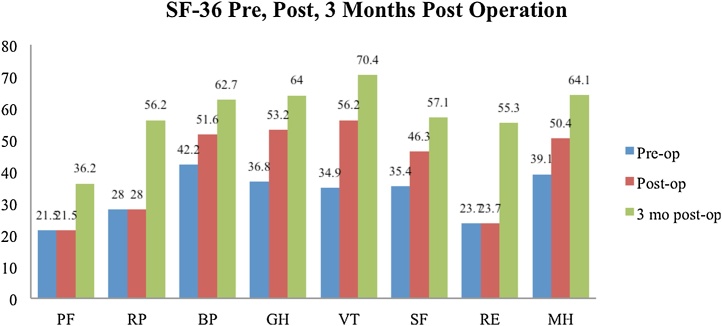
Graph depicting the SF-36 score at pre-, immediately post-, and 3 months post-operatively in the second case. Mean SF-36 score preoperatively were 28.4 for physical condition, 37.3 for mental condition and improved to 48.6 for physical condition and 67.2 for mental condition 3 months after surgery.

Surgical intervention was performed by experienced foot and ankle orthopaedic surgeon accompanied by senior resident with 4 years of specialized training. Post-operative instructions for the patients were non-weight bearing activity and routinely check-up to maintain the condition including clinical and radiological examination. After surgery we were able to maintain a good stability, plantigrade ankle and painless foot on both patients. Patients gave a mean score 9 for satisfaction with the procedure on a scale. Preoperative VAS score was 4 and 3 months postoperative VAS score was 1. AOFAS ankle hindfoot scale pre operative was 58 with two-month post operative score was 83 out of 100. Meanwhile mean SF-36 score preoperatively were 28.4 for physical condition, 37.3 for mental condition and improved to 48.6 for physical condition and 67.2 for mental condition 3 months after surgery (Fig. 5). Postoperatively our patients did not have severe complication observed.

## 3 Discussion

The charcot foot is often described as painless, however patient usually complain mild pain and discomfort despite severe deformity. It is postulated that minor trauma may trigger an inflammatory cascade through a complex pathway such observed in this case where a precipitating trauma happened more than 5 years prior [7].

Trauma may lead to microfracture, subluxation or dislocation creating an abnormal joint which loading is exacerbated by the neuropathy due to the partial or complete lack of pain which cause the continuation of weight bearing in patient [7].

The forefoot is the mostly affected site of the CN foot due to its function as a lever. Hindfoot presentation of CN foot was only 10% comparing to forefoot and midfoot [7]. The other manifestation of charcot in the foot is described based on its anatomy in Table 1.

**Table 1:** Charcot arthropathy anatomical classification.

Pattern	Location	Description
I	Forefoot	Involving the interphalangeal joints, phalanges, metatarsophalangeal joints, and/or distal metatarsal bones; commonly occurring pattern, also seen with plantar ulcerations; seen as osteopenia, osteolysis, juxtaarticular cortical bone defects, subluxation, and destruction on radiographs
II	Tarsometatarsal joints	Involving the tarsometatarsal joints and metatarsal bases, cuneiforms, and cuboid; commonly occurring pattern, with greater frequency in diabetic patients with leprosy; may be associated with plantar ulceration at the apex of deformity; seen as subluxation or fracture-dislocation, collapse of midfoot, and resultant rocker-bottom foot deformity (consistent with initial features of osteoarthritis) on radiographs; may have dorsal prominence at metatarsal bases; late changes include fragmentation
III	Naviculocuneiform, talonavicular, and calcaneocuboid joints	Involving usually the naviculocuneiform joint and navicular bone but also the other midtarsal joints and bones; ulceration may occur at the apex of deformity and may be in combination with pattern II; on radiographs, seen as osteolysis of naviculocuneiform joints with fragmentation; with osseous debris both dorsally and plantarly
IV	Ankle and subtalar joints	Involving the ankle joint with or without the subtalar joint and medial or lateral malleolar fracture; considered a severe structural deformity with instability, may even be associated with minor ankle sprain; on radiographs, seen as malleolar fractures, erosion of bone and cartilage with collapse of joint, free bodies in ankle, extensive destruction, and lateral dislocation of ankle
V	Calcaneus	Rarely involving only the calcaneus bone and usually involving an avulsion fracture of the posterior tubercle; although no joints is involved, the pattern develops in patients with Charcot arthropathy; on radiographs, seen as osteolytic changes in posterior tubercle may ensue; osteolytic changes may also occur at the naviculocuneiform joint due to additional stress during lift odd in the gait cycle (this may be due to lack of an Achilles tendon attachment to the calcaneus)

Chou et al. [8] recently published a multicentre retrospective study of 55 patients (56 ankles) who underwent simultaneous tibiotalocalcaneal arthrodesis with severe disease involving the ankle and subtalar joints. Fusion was achieved in 48 ankles, with an average time to fusion of 19 weeks [9].

Intramedullary fixation is biomechanically more rigid than crossed lag screws when examining flexion and torsional forces [10]. In several biomechanical studies, the IM nail has been shown to provide stability similar to other forms of fixation. IM nails have become a useful device to obtain stability in the foot and ankle.

Our patient came to orthopaedic department with failed fusion after arthrodesis, foot deformity, and stiffness of the ankle. They were primarily diagnosed as Charcot Osteo-neuropathy Brodsky type 2 and 3 A. Based on the diagnosis, we utilized the advantage of ETN nail texture to ease the operation, length of the ETN nail to prevent stress fracture that common site is at the proximal locking screw because of inadequate or too short nail, ETN advanced locking screw to increased stability and preserve soft tissue, and achilles tendon lengthening to restored calcaneal inclination and release stiffness [11]. Chraim et al. [12] stated that the use of retrograde intramedullary compression nail results in good rates of limb salvage when used for hindfoot reconstruction in patients with Charcot arthropathy. In this study, after the surgery already done, we had good foot on plantigrade position, recover foot deformity, osseous stability and reduced pain. It showing the main surgical goals of the tibiotalocalcaneal arthrodesis was achieved. It was the first time to used ETN for ankle arthrodesis with retrograde IM nail of Charcot Osteo-neuropathy Brodsky type 2 and 3 A (Hindfoot Charcot Foot).

AOFAS ankle-hindfoot scale pre operative was 58 with three-month post operative score was 83 out of 100 (Table 1). Generally, the mental condition values improved better by 29.9 compared to physical condition 20.2 which might be due to the length of follow up observed in this case. Nevertheless, postoperatively our patient had wound dehiscence on the lateral border of his ankle which needed to be observed in a longer time also.

All these patients treated with this procedure successfully were because they could tolerate and cooperate with the team, including the surgeons, internists, nurses, and rehabilitation medicine staffs. Subjectively, all two patients were satisfied with the result and they all could do their activity back as before with minimum pain.

## 4 Conclusion

We used ETN nail texture to ease the operation, length of the ETN nail to prevent stress fracture that common site is at the proximal locking screw because of inadequate or too short nail, ETN advanced locking screw to increased stability and preserve soft tissue, and achilles tendon lengthening to restored calcaneal inclanation and release stiffness. The result is good and we achieved the main surgical goals of this technic.

## Conflicts of interest

All authors declare that no conflict of interest in formulating this article.

## Funding

All funding were provided by the authors.

## Ethical approval

This is case report study, no ethical approval were needed. In the other hand, all patient had been informed and gave their consent regarding this publication.

## Consent

All patient had been informed and gave their consent regarding this publication.

## Author contribution

ARBA contributed in conceptualization, data curation, funding acquisition, and writing - original draft.

IO contributed in conceptualization, data curation, funding acquisition, and writing - review & ending.

## Registration of research studies

researchregistry4669.

## Guarantor

The guarantor of this study is IO.

## Provenance and peer review

Not commissioned, externally peer reviewed.
